# Reprogramming the Constant Region of Immunoglobulin G Subclasses for Enhanced Therapeutic Potency against Cancer

**DOI:** 10.3390/biom10030382

**Published:** 2020-03-01

**Authors:** Tae Hyun Kang, Sang Taek Jung

**Affiliations:** 1 Biopharmaceutical Chemistry Major, School of Applied Chemistry, Kookmin University, Seoul 02707, Korea; thkang@kookmin.ac.kr; 2 Department of Biomedical Sciences, Graduate School of Medicine, Korea University, Seoul 02841, Korea

**Keywords:** immunoglobulin G, therapeutic antibodies, Fc receptors, cancer therapy

## Abstract

The constant region of immunoglobulin (Ig) G antibodies is responsible for their effector immune mechanism and prolongs serum half-life, while the fragment variable (Fv) region is responsible for cellular or tissue targeting. Therefore, antibody engineering for cancer therapeutics focuses on both functional efficacy of the constant region and tissue- or cell-specificity of the Fv region. In the functional aspect of therapeutic purposes, antibody engineers in both academia and industry have capitalized on the constant region of different IgG subclasses and engineered the constant region to enhance therapeutic efficacy against cancer, leading to a number of successes for cancer patients in clinical settings. In this article, we review IgG subclasses for cancer therapeutics, including (i) IgG1, (ii) IgG2, 3, and 4, (iii) recent findings on Fc receptor functions, and (iv) future directions of reprogramming the constant region of IgG to maximize the efficacy of antibody drug molecules in cancer patients.

## 1. Introduction

**Figure 1:**
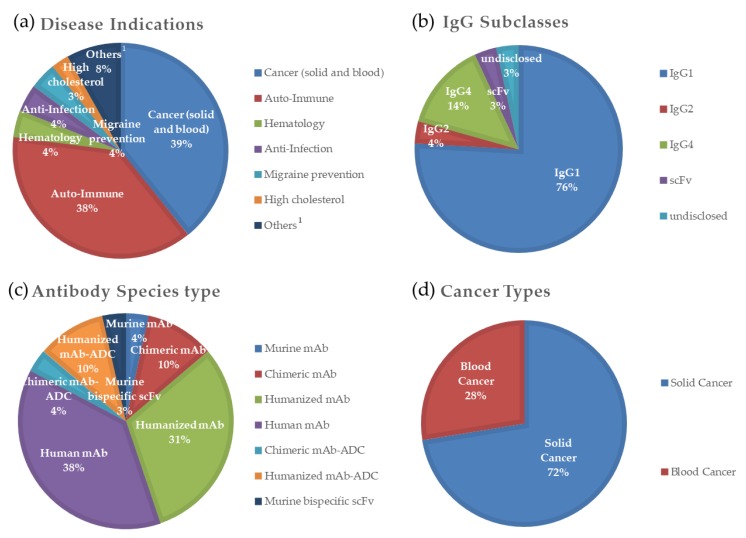
Indication and molecular types of therapeutic antibodies approved by the US FDA and EMA, classified by (**a**) disease indications of all 74 therapeutic antibodies; (**b**) antibody subclasses of 29 antibodies for cancer; (**c**) species type of 29 antibodies for cancer, i.e., murine, chimeric, humanized, or fully human; and (**d**) cancer types of 29 antibodies for cancer (blood or solid cancer). These figures were classified using data from “The Antibody Society (2020)” [2]. ^1^ Others in panel (**a**) include prevention of kidney transplant, macular degeneration, Muckle–Wells syndrome, bone loss, high cholesterol, X-linked hypophosphatemia, and osteoporosis in postmenopausal women at increased risk of fracture.

Therapeutic monoclonal antibodies (mAbs) comprised more than 70% of global biologics revenue in 2018 [1]. Up to 21 January 2020, the US Food and Drug Administration (FDA) and European Medicines Agency (EMA) approved 74 therapeutic antibodies, 29 of which were for cancer-related disease (39%) (Figure 1a). Interestingly, IgG1 constitutes at least 22 of the 29 cancer-related therapeutic antibodies, while all other IgG subclass antibodies, including IgG4 but not panitumumab (IgG2), were approved in 2014 or later (Figure 1b) [2]. As of January 2020, human or humanized antibodies constituted more than 70% of the total cancer-related antibodies (Figure 1c). Among the 29 antibodies, 8 were for blood cancer and the other 21 were for solid cancer treatment (Figure 1d), including breast cancer, colorectal cancer, head and neck cancer, lung cancer, rectal cancer, glioblastoma, melanoma, myeloma, neuroblastoma, and sarcoma [2,3].

It is undeniable that antibody therapies have greatly improved the survival rate of cancer patients, and they are considered one of the most effective disease-targeting moieties for cancer. This is because therapeutic antibodies selectively target cancerous cells or immune leukocytes and thus exhibit lower toxicity compared to conventional small molecule-based chemotherapy or radiotherapy. However, treatments with mAbs rarely lead to complete recovery from diseases [4]. Therefore, mAbs require combination with other toxic therapeutic modalities [5,6,7], despite their extraordinary specificities for cancer tissue or immune leukocytes. When they directly target tumor associated antigens (TAAs) [8,9,10,11], the native effector function of mAbs may not be sufficient to eradicate refractory tumor cells, and tumor cells may be resistant to immune effector cells when mAbs target and activate immune leukocytes [12,13,14,15,16]. This necessitates understanding of the antibody effector mechanism underlying Fc–Fc receptor biology and obliges antibody engineers to investigate the effector functions of mAbs with desired constant regions.

As of January 2020, all FDA- and EMA-approved immunoglobulin isotypes are IgG antibodies [2,18]. These antibodies bind to the family of Fc receptors (Fc gamma receptors, FcγRs) to activate or inhibit signaling that mediates complex immune responses upon formation of immune complexes [19,20]. The FcγRs bind IgGs [21,22,23] to initiate and regulate various effector functions, such as inhibition of B cell proliferation, phagocytosis, degranulation of cytotoxic molecules in granulocytes, and cytokine production [24,25]. Three different classes of human Fc gamma receptors for IgG have been identified: FcγRI (CD64), FcγRII (CD32), and FcγRIII (CD16) [26]. Among FcγRs, FcγRI exhibits high affinity (K_D_ = 10^−8^ M) to the antibody-constant region, whereas FcγRII and FcγRIII display low affinity (K_D_ = 10^−6^~10^−7^ M) to the fragment crystallizable (Fc) region of IgG [27]. Mechanistically, FcγRs are grouped into two groups: activating FcγRs such as FcγRI (CD64), FcγRIIa (CD32a), and FcγRIIIa (CD16a), and the inhibitory FcγR, FcγRIIb (CD32b). These activating and inhibitory FcγRs transduce their functional signaling through immunoreceptor tyrosine-based activation motif (ITAM) or inhibitory motif (ITIM), respectively [20,21].

Human FcγRI (CD64) is a glycoprotein that binds to monomeric IgG with high affinity (K_D_ = 10^−8^~10^−9^ M). Human FcγRI binds with high affinity to IgG1 and IgG3 but binds weakly to IgG4 and very weakly with IgG2 (Table 1) [28]. FcγRI consists of an α-chain with multiple glycan chains and two γ-chain subunits of FcεRI [29,30].

FcγRII is expressed more frequently on cells of hematopoietic lineage compared to other FcγRs. There are three different FcγRII isoforms: FcγRIIa, FcγRIIb, and FcγRIIc. Whereas FcγRIIa and FcγRIIc transmit activating signals on immune leukocytes, FcγRIIb produces inhibitory signals throughout the membranes of blood cells. FcγRIIa exhibits diverse expression profiles on macrophages, monocytes, neutrophils, and platelets. Relative to FcγRIIa, FcγRIIc has been reported to be expressed less frequently, though it is uniquely present on natural killer (NK) cells [31]. FcγRIIa is generally thought to be responsible for phagocytosis, cytotoxicity, and inflammatory cytokine release, but these mechanisms remain to be validated. Unlike FcγRIIa, FcγRIIb can be found on basophils, mast cells, monocytes, macrophages, dendritic cells (DC), and even B cells. FcγRIIb down-regulates immune activating signals by flanking the triggered activating FcγRs [32].

There are two FcγRIII isoforms identified so far: FcγRIIIa and FcγRIIIb. FcγRIIIb is expressed on neutrophils, and activation of FcγRIIIb stimulates degranulation and production of reactive oxygen intermediates (ROI) in neutrophils [33]. FcγRIIIa is well-known for its contribution to ADCC activity because it is the only FcγR expressed on NK cells. It is also regarded as an initiator for endocytosis, phagocytosis, and cytokine production of existing immune leukocytes; however, the exact implications are still unknown. The downstream signal transduction triggered by FcγRIIIa as well as FcγRI requires association of γ or ζ chain on the cell surface [34,35].

FcγRs are important in mediation of both humoral immunity and immunologic responses [36]. The functions of Fc receptors must be elucidated to establish a direction for reprogramming the constant regions of IgG antibodies for therapeutic purposes. However, it is not possible to elucidate the function of FcγRs in various human leukocytes with irregular FcγRs profiles [37] without FcγR-selective Fc.

Despite the negligence of FcγRs function, researchers in academia and biopharmaceuticals have extensively investigated constant regions of IgG subclasses and even engineered them to maximize therapeutic efficacy in cancer therapy. In this paper, we review advantages and disadvantages of IgG subclasses for therapeutic usage, including i) IgG1, ii) IgG2, 3, and 4, based on known molecular properties and affinities to Fc receptors, iii) recent findings on Fc receptor functions for antibody cancer therapy, and finally iv) future directions of reprogramming the constant region of the IgG antibody for better cancer therapy.

## 2. IgG1: The Most Abundant IgG Antibody in Cancer Therapeutics

IgG1 shows the highest serum abundance (65% of total IgG) [38] and is the most commonly used therapeutic IgG antibody among IgG subclasses [2] because of its (i) significant binding to FcγRs, (ii) short hinge length (15 amino acid residues) with two inter-heavy chain disulfide bonds, enabling relatively facile bioprocessing, and (iii) long serum half-life (Table 1). One immunosurveillance mechanism in cancer involves generation of IgG antibodies that surround tumor cells via affinities to cancer antigens. Especially, cancer-specific IgG1 antibodies attract diverse immune cells to the cancer site by Fc–FcγRs interactions, while IgG2 or 4 antibodies (25% of total IgG) barely do (Table 1) [17]. Activated leukocytes expressing FcγRs on their plasma membranes can target tumor cells by releasing cytotoxic granules or inducing phagocytosis, processes called antibody-dependent cell-mediated cytotoxicity (ADCC) or antibody-dependent cell-mediated phagocytosis (ADCP), respectively [39]. Moreover, IgG1 antibodies can maximize these antibody-mediated activities by Fc-engineering methodologies to enhance efficacy. Engineering Fc domains with enhanced affinity for activating FcγRs relative to the inhibitory FcγRIIb has been attempted to strengthen ADCC or ADCP activity [40,41].

In the past 15 years, Fc engineering in academia and biotechnology has been directed toward enhancing known activating FcγRs, such as FcγRIIa (i.e., major contributor to ADCP activity) and FcγRIIIa (i.e., major contributor to the NK ADCC activity of NK cells), relative to the inhibitory FcγRIIb. For example, the biotech company Xencor (CA, USA) succeeded in engineering Fc with increased FcγRIIIa binding relative to FcγRIIb compared to the endogenous human Fc. One of their mutants, S239D/I332E/A330L (EU numbering), exhibits a more than 100-fold increase in FcγRIIIa binding and significantly higher ADCC activity compared to wild-type IgG Fc [42]. A distinctive Fc mutant from the same company, G236A, resulted in increased FcγRIIa over FcγRIIb affinity with notable ADCP activity relative to the native Fc [43]. Another biotech company, MacroGenics (MD, USA), isolated an Fc variant mutant, L235V/F243L/R292P/Y300L/P396L, with increased FcγRIIIa over FcγRIIb binding ratio relative to the wild-type Fc [44]. As expected, IgG antibody with mutant Fc exhibited improved ADCC activity with human peripheral blood mononuclear cells (PBMC) and NK cells as well as tumor regression in a human FcγRIIIa-transgenic mice model [45]. Further, the company submitted a Biologics License Application (BLA) for FDA approval on December 2019 with successful phase 3 clinical trial with margetuximab, an mAb that adapted the Fc variant to Fab targeting Her2. Their results showed a 24% risk reduction in patients on margetuximab compared to trastuzumab in 536 patients with breast cancer [46]. Another Fc-engineered anti-CD20 antibody, ocaratuzumab, which exhibits 6-fold increased ADCC activity relative to rituximab, is being developed by Mentrik Biotech and is under phase 3 clinical trial for patients with relapsed follicular lymphoma and other oncology indications [47].

Antibody engineers have focused on using amino acid mutations to develop an IgG1 antibody Fc with enhanced affinity for FcγRIIIa, resulting in potentiated effector function [44,48,49,50,51,52,53,54]. Meanwhile, researchers in the field of glycan engineering have worked to maximize the affinity of IgG1 Fc to FcγRIIIa by modifying Fc glycans such as fucose [55] and branching N-acetylglucosamine (GNAc) [56,57,58,59,60]. This is because the Asn297-linked carbohydrate chains on the Fc region of IgG1 are critical for FcγRs binding [61,62,63]. Removal of fucose on IgG1 Fc carbohydrate significantly increased FcγRIIIa affinity, resulting in enhanced ADCC and ADCP activity relative to the native IgG1 Fc counterpart [64]. In January 2019, the US FDA approved obinutuzumab, an afucosylated anti-CD20 antibody created by scientists at GlycArt Biotechnology, in combination with ibrutinib for first-line treatment in patients with chronic lymphocytic leukemia/small lymphocytic lymphoma [5,65]. Another example is a humanized afucosylated anti-CCR4 antibody, mogamulizumab (trade name Poteligeo), which was approved by the FDA for cutaneous T cell lymphoma (CTCL) in August 2018 [66]. These successes in oncology drug approval indicate the significance of potentiated IgG1 Fc functions for cancer therapeutics.

## 3. IgG2 and 4 with Relatively Lower Effector Functions; IgG3 with a Long Hinge Region Compared to IgG1

As mentioned above, IgG1 mAbs or antibodies with enhanced tumor-killing functions of Fc can be advantageous over native Fc when they directly target TAAs. However, there are cases where target cell-clearance activities such as ADCC, ADCP, and/or CDC can be detrimental. For example, mAbs may act as immune-checkpoint blockers or bispecific leukocyte engagers to target tumor tissues. In these cases, constant regions with fewer effector functions are favorable [67,68]. To retain immune leukocytes that kill tumor cells, it is important to minimize the effector function of Fc so that immunosurveillance can be turned on, because FcγR activation thresholds vary among patients. One simple way to lower FcγR engagement is to employ the constant region of IgG2 or IgG4 because of its relatively lower FcγR affinity, compared to that of IgG1 (Table 1) [17,41].

IgG2 has minimal FcγRIIa affinity relative to IgG1 (Table 1) and can elicit ADCC [69] and ADCP [70] by monocytes and macrophages, respectively. Because IgG2 can form four inter-heavy chain disulfide bonds (Table 1) [71] with three possible IgG2 disulfide isoforms [72], a super-agonistic property can be achieved by changing conformation of the hinge region of IgG2 [73]. The hinge region of IgG2 is most resistant to proteolysis among IgG subclasses [74]. As of January 2020, panitumumab is the only IgG2 therapeutic antibody approved by the US and EU.

IgG4 has relatively low affinity to FcγRI and FcγRII (Table 1) [17] and can elicit ADCP by macrophages [70] but not ADCC by NK cells [75]. IgG4 can undergo Fab-arm exchange, which results in native bispecific antibodies [76]; this has been observed in vivo [77,78]. This phenomenon can be abolished by introducing an S228P mutation that abrogates formation of intra-chain disulfide bond isomers [77,79]. Currently, the three anti-PD-1 drugs nivolumab, pembrolizumab, and cemiplimab are on US and EU markets, approved for head and neck cancer, advanced melanoma, and cutaneous squamous cell carcinoma, respectively. These antibodies share an IgG4 constant region with the S228P mutation, targeting effector T lymphocytes with relatively lower effector Fc functions compared to the IgG1 constant region (Table 1).

IgG3 shows the highest affinity to all the FcγRs among the IgG subclasses [17] but has a short serum half-life (Table 1) compared to IgG1 due to R435, which affects neonatal Fc receptor (FcRn) binding. This therapeutic disadvantage can be overcome by R435H mutation, which is present in IgG1, 2, and 4 [80]. The main reason why there are not yet clinically available IgG3 antibodies is its 62-amino-acid long hinge region with 11 inter-heavy chain disulfide bonds (Table 1) [17], which require complex biomanufacturing and bioprocessing.

## 4. Recent Findings of Fc Receptor Functions for Treating Malignancy

A well-established function of FcγRIIIa, capable of engaging NK cells and inducing ADCC activity, highlights the superior therapeutic efficacy of mAbs with Fcs engineered for higher FcγRIIIa affinity relative to native IgG1 Fc [81,82]. Contradictorily, other studies reported that exhaustion of NK cells did not significantly lower the therapeutic efficacy of anti-CD20 mAbs [83]. Rather, macrophage depletion significantly reduced the tumor regression activity of mAbs targeting CD20 [83], CD30 [84], and CD40 [85] in mice. Together with well-known functions of macrophages in tumor phagocytic activity [86], these results indicate that ADCP activity is critical in therapeutic efficacy against cancer. The ADCP activity of macrophages is triggered by FcγRIIa intracellular signaling [87,88,89]; however, anti-CD20 or anti-Her2 antibodies with engineered Fcs that only bind to FcγRIIIa triggered not only ADCC, but also ADCP using human NK cells and macrophages in vitro, which is an unknown function of FcγRIIIa [90]. This study again highlights a clinical ramification of FcγRIIIa in cancer therapeutics.

The role of FcγRs in cancer therapeutics calls attention to mAbs targeting both TAAs and immune-checkpoint receptors on leukocytes. FcγRIIb is highlighted as an immune-checkpoint receptor in various immune cells and is considered an “antibody checkpoint” in cancer immunotherapy [91]. Furthermore, the experimental result that mice injected with Fc-engineered antibodies for enhanced FcγR affinity exhibited survival even after re-challenge with tumor cells indicates that FcγR may contribute to long-term antitumor T-cell memory immunity responses [92].

When the inhibitory FcγRIIb is genetically knocked out, the therapeutic efficacy of mAbs targeting CD20, Her2, and EGFR is significantly enhanced in hematologic malignancy and solid cancers [40]. This indicates that FcγRIIb is an immune-checkpoint molecule similar to CTLA-4 or PD-1 in T cells [4,12,93]. Interestingly, FcγRIIb on B cells limits the anti-tumor activity of the anti-CD20 antibody rituximab [94] and promotes internalization of the rituximab antibody [95]. This FcγRIIb-mediated internalization of rituximab was correlated with receptor expression in different subtypes of B cell lymphoma, such as chronic lymphocytic leukemia (CLL), mantle cell lymphoma (MCL), marginal zone lymphoma, follicular lymphoma (FL), and diffuse large B cell lymphoma [96,97]. Therefore, FcγRIIb seems to limit the potency of therapeutic antibody and promotes antibody drug resistance. Developing anti-FcγRIIb antibodies specifically blocking the rituximab-FcγRIIb interaction [98] and using them in combination with rituximab may be a decent strategy to overcome anti-CD20 drug resistance in the clinic.

**Figure 2:**
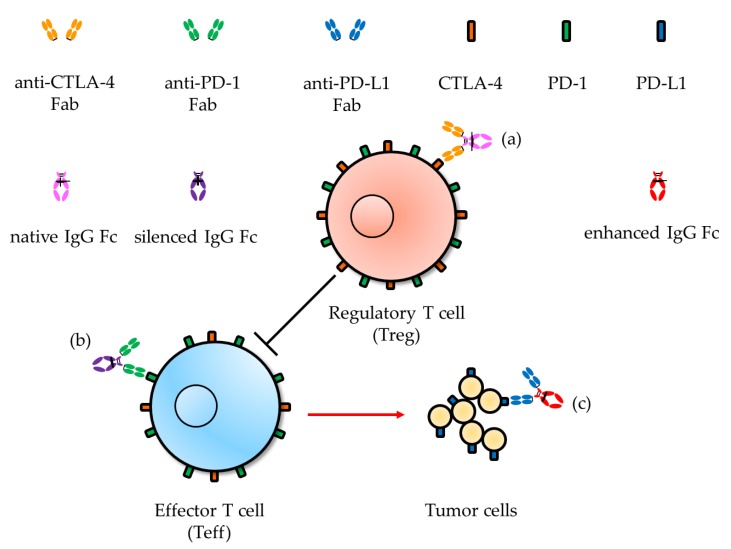
Proposed Fc function on immune-checkpoint blocking antibodies targeting (**a**) CTLA-4, with FcγR-mediated clearance of regulatory T (Treg) cells; (**b**) PD-1, with minimized antibody-dependent cell-mediated cytotoxicity (ADCC) or antibody-dependent cell-mediated phagocytosis (ADCP) activity on effector T (Teff) cells; (**c**) PD-L1, with enhanced ADCC or ADCP activity on tumor cells.

Anti-CTLA-4 antibody is an immune checkpoint blocker associated with improved survival in melanoma patients having the high-affinity FcγRIIIa-V158 allele to IgG Fc relative to those carrying the low affinity allele, FcγRIIIa-F158 [99]. This clinical outcome of anti-CTLA-4 was expected because FcγR-mediated clearance of regulatory T (Treg) cells, which express notably higher levels of CTLA-4 than effector T (Teff) cells, is favorable relative to that of Teff cells (Figure 2a). In the PD-1/PD-L1 axis, anti-PD-1 antibody bearing constant region of IgG1 with higher FcγR affinity exhibited lower therapeutic efficacy, compared to that having constant region of IgG4 [100]. This indicates that FcγR negatively regulates anti-PD-1 antibody therapy due to ADCC or ADCP activity on Teff cells (Figure 2b). Conversely, the therapeutic efficacy of anti-PD-L1 antibody was enhanced with the constant region of IgG1 relative to IgG4 (Table 1) due to ADCC or ADCP activity targeting PD-L1-expressing tumor cells (Figure 2c) [101].

## 5. Future Directions for Reprogramming the Constant Region of IgG Antibodies for Treating Malignancy

There have been beneficial clinical outcomes of Fc-engineered antibodies with enhanced affinity to FcγRIIIa relative to native IgG1 Fc counterparts, including margetuximab [46], ocaratuzumab [47], obinutuzumab [65], and mogamulizumab [66]. However, it is not clear which leukocytes or FcγRs are responsible for the potentiated therapeutic results because various immune cells exhibit discrete FcγR expression profiles [102]. In addition, every IgG1 Fc variant reprogrammed for improved affinity toward FcγRIIIa also shows higher affinity to other FcγRs compared with wild-type IgG1 Fc. It not well documented which FcγRs contribute to clinical benefits, especially when considering macrophages or dendritic cells in contrast to NK cells that only express FcγRIIIa (CD16a) [102]. This is important because intra-tumoral macrophages [95] and dendritic cells are critical as anti-tumoral immune effectors [103].

**Figure 3:**
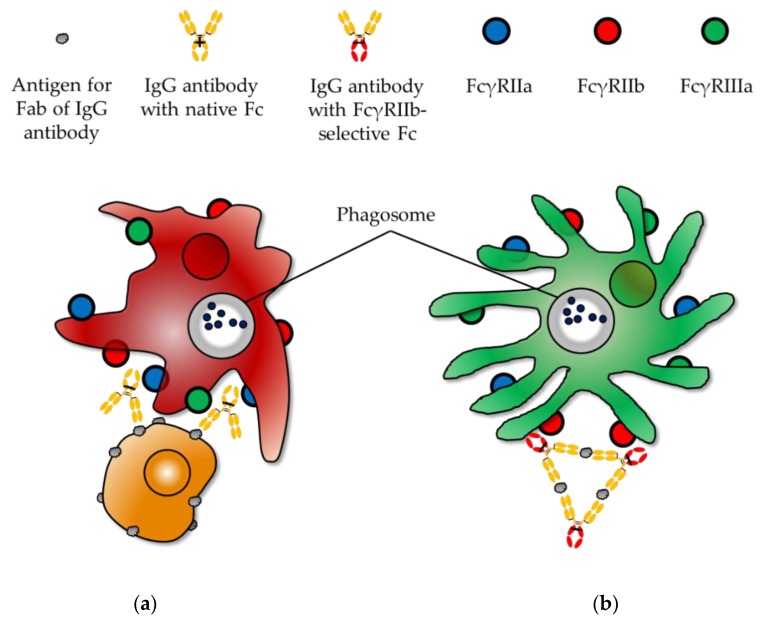
To elucidate Fc function, FcγR-selective Fc should be present. (**a**) Native IgG antibodies engage all types of FcγRs, making it very difficult to dissect the function of each FcγR in macrophages until the FcγR-selective IgG antibody is present; (**b**) FcγRIIb function in dendritic cells can only be clarified in the presence of FcγRIIb-selective IgG antibody.

FcγRIIa and FcγRIIb are expressed relatively highly in macrophages and dendritic cells compared to other FcγRs [99]. Moreover, FcγRIIa is the only activating FcγR on the plasma membrane of human dendritic cells, while FcγRIIb on the same cells regulates antigen presentation in collaboration with FcγRIIa [104]. Therefore, it is critical to define the functions of FcγRIIa and FcγRIIb on various types of immune cells, especially macrophages and dendritic cells. For this purpose, an Fc that selectively binds to FcγRIIa and FcγRIIb is essential (Figure 3). FcγR-selective antibodies are advantageous over blocking antibodies or siRNAs because they will elucidate direct functions of antibody-mediated activity rather than just providing indirect evidence by inhibiting intracellular signal cascades; however, neither FcγRIIa- nor FcγRIIb-selective Fc is available at present. Engineering of FcγR-selective Fc may be challenging, as the amino acid sequences of FcγR ecto-domains are highly homologous (FcγRIIa and FcγRIIb exhibit 96% identity in amino acid sequence) [105].

With the precedent of FcγRI- and FcγRIIIa-selective Fcs [90,106], FcγRIIa- and FcγRIIb-selective Fcs should be developed to dissect antibody-mediated effector mechanisms for understanding human immunobiology and providing future antibody therapeutics. These selective Fcs are designed for (i) tumor cell destruction, such as anti-CD20 antibodies [5], (ii) immune cell activation, such as agonistic anti-CD40 [107,108] or anti-TNFR antibodies [109], which elicit antigen presentation and subsequent adaptive immune response by engaging FcγRIIb, and (iii) anti-tumor vaccine effect [92]. FcγRs have been demonstrated to contribute to the adaptive memory immune response. However, it is not possible to determine which FcγRs are responsible for long-term anti-tumor immunity until full sets of FcγRs-specific Fcs are present.

Now that mouse models in which not only the murine FcγR genes have been exchanged with human counterparts [110,111] but also the murine μ region of heavy chain and kappa regions of Ig light chain genes have been exchanged with human IgG constant heavy regions and human κ light region genes, respectively [112], are available, FcγRs-specific IgG antibodies can be developed to indicate the contribution of FcγR to long-term anti-tumor efficacy using these mouse models. This work has previously been evaluated [92] but since IgG antibodies used in the study were not strictly FcγRs-specific, we cannot draw any conclusion on which FcγR is responsible for long-term anti-cancer efficacy. The animal models can be very useful to elucidate the FcγR function in adaptive immunity; however, they have limitations in the immunobiology of a mouse, such that murine FcγR expression profiles on murine immune leukocytes cannot be translated into that of humans.

## 6. Conclusions

Capitalizing on native IgG1 subclass or engineered constant regions of IgG is a potent strategy used to potentiate therapeutic efficacy that has had several clinical successes, including approval of antibody drugs with significant or even improved affinity to activating FcγRs. On the contrary, IgG2, IgG4, or even mutant constant regions to silence effector functions are required for antibody cancer therapy, especially when a drug targets immune-checkpoint molecules on effector leukocytes. However, it is not yet clear which FcγRs and which immune effector cells are responsible for anti-tumor efficacy. FcγR-selective Fcs are prerequisites for this elucidation, as antibodies that selectively activate each FcγR will directly gain immune function. Once the function of an IgG constant region becomes clear by Fc reprogramming, antibody platforms can be developed to broaden therapeutic windows for cancer treatment.

## Figures and Tables

**Table 1:** Molecular properties of IgG antibody subclasses.

IgG Subclasses	Hinge Length (Amino Acid Residues)	Number of Disulfide Bonds in the Hinge Region	Serum Half-Life (Week)	Relative Affinities to FcγRs ^2^ (Expected Effector Functions via FcγRs)					
				I	IIa	IIb	IIc	IIIa	IIIb
IgG1	15	2	3	+++	+++	+	+	++	+++
IgG2	12	4 ^1^	3	-	++	-	-	-/+	-
IgG3	62 ^1^	11 ^1^	1	++++	++++	++	++	++++	++++
IgG4	12	2	3	++	++	++	++	-	-

^1^ Values differ among antibody allotypes. ^2^ Values were adapted from IgG immune complex (IC) binding to FcγR-transfected cells using FACS analysis in Bruhns et al. (2009) [17].

## References

[1] (2019). Blockbuster Biologics 2018: Sales of Recombinant Therapeutic Antibodies & Proteins. LMCA0175.

[2] The Antibody Society. https://www.antibodysociety.org/resources/approved-antibodies/.

[3] Grilo A.L., Mantalaris A. (2019). The increasingly human and profitable monoclonal antibody market. Trends Biotechnol..

[4] Sharma P., Hu-Lieskovan S., Wargo J.A., Ribas A. (2017). Primary, Adaptive, and Acquired Resistance to Cancer Immunotherapy. Cell.

[5] Goede V., Fischer K., Busch R., Engelke A., Eichhorst B., Wendtner C.M., Chagorova T., de la Serna J., Dilhuydy M.S., Illmer T. (2014). Obinutuzumab plus chlorambucil in patients with CLL and coexisting conditions. N. Engl. J. Med..

[6] Baselga J., Cortes J., Kim S.B., Im S.A., Hegg R., Im Y.H., Roman L., Pedrini J.L., Pienkowski T., Knott A. (2012). Pertuzumab plus trastuzumab plus docetaxel for metastatic breast cancer. N. Engl. J. Med..

[7] Gopal A.K., Kahl B.S., de Vos S., Wagner-Johnston N.D., Schuster S.J., Jurczak W.J., Flinn I.W., Flowers C.R., Martin P., Viardot A. (2014). PI3Kdelta inhibition by idelalisib in patients with relapsed indolent lymphoma. N. Engl. J. Med..

[8] Cheson B.D., Leonard J.P. (2008). Monoclonal antibody therapy for B-cell non-Hodgkin’s lymphoma. N. Engl. J. Med..

[9] Gradishar W.J. (2012). HER2 therapy—An abundance of riches. N. Engl. J. Med..

[10] Jonker D.J., O’Callaghan C.J., Karapetis C.S., Zalcberg J.R., Tu D., Au H.J., Berry S.R., Krahn M., Price T., Simes R.J. (2007). Cetuximab for the treatment of colorectal cancer. N. Engl. J. Med..

[11] Lokhorst H.M., Plesner T., Laubach J.P., Nahi H., Gimsing P., Hansson M., Minnema M.C., Lassen U., Krejcik J., Palumbo A. (2015). Targeting CD38 with Daratumumab Monotherapy in Multiple Myeloma. N. Engl. J. Med..

[12] Larkin J., Chiarion-Sileni V., Gonzalez R., Grob J.J., Cowey C.L., Lao C.D., Schadendorf D., Dummer R., Smylie M., Rutkowski P. (2015). Combined Nivolumab and Ipilimumab or Monotherapy in Untreated Melanoma. N. Engl. J. Med.

[13] Ribas A., Puzanov I., Dummer R., Schadendorf D., Hamid O., Robert C., Hodi F.S., Schachter J., Pavlick A.C., Lewis K.D. (2015). Pembrolizumab versus investigator-choice chemotherapy for ipilimumab-refractory melanoma (KEYNOTE-002): A randomised, controlled, phase 2 trial. Lancet Oncol..

[14] Robert C., Ribas A., Wolchok J.D., Hodi F.S., Hamid O., Kefford R., Weber J.S., Joshua A.M., Hwu W.J., Gangadhar T.C. (2014). Anti-programmed-death-receptor-1 treatment with pembrolizumab in ipilimumab-refractory advanced melanoma: A randomised dose-comparison cohort of a phase 1 trial. Lancet.

[15] Robert C., Long G.V., Brady B., Dutriaux C., Maio M., Mortier L., Hassel J.C., Rutkowski P., McNeil C., Kalinka-Warzocha E. (2015). Nivolumab in previously untreated melanoma without BRAF mutation. N. Engl. J. Med..

[16] Weber J.S., D’Angelo S.P., Minor D., Hodi F.S., Gutzmer R., Neyns B., Hoeller C., Khushalani N.I., Miller W.H., Lao C.D. (2015). Nivolumab versus chemotherapy in patients with advanced melanoma who progressed after anti-CTLA-4 treatment (CheckMate 037): A randomised, controlled, open-label, phase 3 trial. Lancet Oncol..

[17] Bruhns P., Iannascoli B., England P., Mancardi D.A., Fernandez N., Jorieux S., Daeron M. (2009). Specificity and affinity of human Fcgamma receptors and their polymorphic variants for human IgG subclasses. Blood.

[18] Brezski R.J., Georgiou G. (2016). Immunoglobulin isotype knowledge and application to Fc engineering. Curr. Opin. Immunol..

[19] Nimmerjahn F., Ravetch J.V. (2008). Fcgamma receptors as regulators of immune responses. Nat. Rev. Immunol..

[20] Ravetch J.V., Lanier L.L. (2000). Immune inhibitory receptors. Science.

[21] Ravetch J.V., Bolland S. (2001). IgG Fc receptors. Annu. Rev. Immunol..

[22] Berken A., Benacerraf B. (1966). Properties of antibodies cytophilic for macrophages. J. Exp. Med..

[23] Takai T. (2005). Fc receptors and their role in immune regulation and autoimmunity. J. Clin. Immunol..

[24] Ivan E., Colovai A.I. (2006). Human Fc receptors: Critical targets in the treatment of autoimmune diseases and transplant rejections. Hum. Immunol..

[25] Cohen-Solal J.F., Cassard L., Fridman W.H., Sautes-Fridman C. (2004). Fc gamma receptors. Immunol. Lett..

[26] Nimmerjahn F., Ravetch J.V. (2006). Fcgamma receptors: Old friends and new family members. Immunity.

[27] Krapp S., Mimura Y., Jefferis R., Huber R., Sondermann P. (2003). Structural analysis of human IgG-Fc glycoforms reveals a correlation between glycosylation and structural integrity. J. Mol. Biol..

[28] van der Pol W.L., van de Winkel J.G. (1998). Immunology in clinical practice. X. IgG receptors: Structure, function and immunotherapy. Ned. Tijdschr. Geneeskd..

[29] Allen J.M., Seed B. (1989). Isolation and expression of functional high-affinity Fc receptor complementary DNAs. Science.

[30] Kuster H., Thompson H., Kinet J.P. (1990). Characterization and expression of the gene for the human Fc receptor gamma subunit. Definition of a new gene family. J. Biol. Chem..

[31] Cassel D.L., Keller M.A., Surrey S., Schwartz E., Schreiber A.D., Rappaport E.F., McKenzie S.E. (1993). Differential expression of Fc gamma RIIA, Fc gamma RIIB and Fc gamma RIIC in hematopoietic cells: Analysis of transcripts. Mol. Immunol..

[32] Phillips N.E., Parker D.C. (1983). Fc-dependent inhibition of mouse B cell activation by whole anti-mu antibodies. J. Immunol..

[33] Salmon J.E., Millard S.S., Brogle N.L., Kimberly R.P. (1995). Fc gamma receptor IIIb enhances Fc gamma receptor IIa function in an oxidant-dependent and allele-sensitive manner. J. Clin. Investig..

[34] Gessner J.E., Heiken H., Tamm A., Schmidt R.E. (1998). The IgG Fc receptor family. Ann. Hematol..

[35] Siberil S., Dutertre C.A., Fridman W.H., Teillaud J.L. (2007). FcgammaR: The key to optimize therapeutic antibodies?. Crit. Rev. Oncol. Hematol..

[36] Huber R., Deisenhofer J., Colman P.M., Matsushima M., Palm W. (1976). Crystallographic structure studies of an IgG molecule and an Fc fragment. Nature.

[37] Bournazos S. (2019). IgG Fc Receptors: Evolutionary Considerations. Curr. Top. Microbiol. Immunol..

[38] French M. (1986). Serum IgG subclasses in normal adults. Monogr. Allergy.

[39] Park H.I., Yoon H.W., Jung S.T. (2016). The Highly Evolvable Antibody Fc Domain. Trends Biotechnol..

[40] Clynes R.A., Towers T.L., Presta L.G., Ravetch J.V. (2000). Inhibitory Fc receptors modulate in vivo cytotoxicity against tumor targets. Nat. Med..

[41] Nimmerjahn F., Ravetch J.V. (2005). Divergent immunoglobulin g subclass activity through selective Fc receptor binding. Science.

[42] Lazar G.A., Dang W., Karki S., Vafa O., Peng J.S., Hyun L., Chan C., Chung H.S., Eivazi A., Yoder S.C. (2006). Engineered antibody Fc variants with enhanced effector function. Proc. Natl. Acad. Sci. USA.

[43] Richards J.O., Karki S., Lazar G.A., Chen H., Dang W., Desjarlais J.R. (2008). Optimization of antibody binding to FcgammaRIIa enhances macrophage phagocytosis of tumor cells. Mol. Cancer Ther..

[44] Stavenhagen J.B., Gorlatov S., Tuaillon N., Rankin C.T., Li H., Burke S., Huang L., Johnson S., Koenig S., Bonvini E. (2008). Enhancing the potency of therapeutic monoclonal antibodies via Fc optimization. Adv. Enzyme Regul..

[45] Nordstrom J.L., Gorlatov S., Zhang W., Yang Y., Huang L., Burke S., Li H., Ciccarone V., Zhang T., Stavenhagen J. (2011). Anti-tumor activity and toxicokinetics analysis of MGAH22, an anti-HER2 monoclonal antibody with enhanced Fcgamma receptor binding properties. Breast Cancer Res..

[46] Taylor N.P. (2019). MacroGenics’ margetuximab beats Herceptin in phase 3. FierceBiotech.

[47] VanDerMeid K.R., Elliott M.R., Baran A.M., Barr P.M., Chu C.C., Zent C.S. (2018). Cellular Cytotoxicity of Next-Generation CD20 Monoclonal Antibodies. Cancer Immunol. Res..

[48] Shields R.L., Namenuk A.K., Hong K., Meng Y.G., Rae J., Briggs J., Xie D., Lai J., Stadlen A., Li B. (2001). High resolution mapping of the binding site on human IgG1 for Fc gamma RI, Fc gamma RII, Fc gamma RIII, and FcRn and design of IgG1 variants with improved binding to the Fc gamma R. J. Biol. Chem..

[49] Oganesyan V., Damschroder M.M., Leach W., Wu H., Dall’Acqua W.F. (2008). Structural characterization of a mutated, ADCC-enhanced human Fc fragment. Mol. Immunol..

[50] Saxena A., Wu D. (2016). Advances in Therapeutic Fc Engineering—Modulation of IgG-Associated Effector Functions and Serum Half-life. Front. Immunol..

[51] Ashoor D.N., Ben Khalaf N., Bourguiba-Hachemi S., Marzouq M.H., Fathallah M.D. (2018). Engineering of the upper hinge region of human IgG1 Fc enhances the binding affinity to FcgammaIIIa (CD16a) receptor isoform. Protein. Eng. Des. Sel..

[52] Zhang D., Goldberg M.V., Chiu M.L. (2016). Fc Engineering Approaches to Enhance the Agonism and Effector Functions of an Anti-OX40 Antibody. J. Biol. Chem..

[53] Jo M., Kwon H.S., Lee K.H., Lee J.C., Jung S.T. (2018). Engineered aglycosylated full-length IgG Fc variants exhibiting improved FcgammaRIIIa binding and tumor cell clearance. MAbs.

[54] Yoon H.W., Jo M., Ko S., Kwon H.S., Lim C.S., Ko B.J., Lee J.C., Jung S.T. (2019). Optimal combination of beneficial mutations for improved ADCC effector function of aglycosylated antibodies. Mol. Immunol..

[55] Shields R.L., Lai J., Keck R., O’Connell L.Y., Hong K., Meng Y.G., Weikert S.H., Presta L.G. (2002). Lack of fucose on human IgG1 N-linked oligosaccharide improves binding to human Fcgamma RIII and antibody-dependent cellular toxicity. J. Biol. Chem..

[56] Li T., DiLillo D.J., Bournazos S., Giddens J.P., Ravetch J.V., Wang L.X. (2017). Modulating IgG effector function by Fc glycan engineering. Proc. Natl. Acad. Sci. USA.

[57] Li W., Zhu Z., Chen W., Feng Y., Dimitrov D.S. (2017). Crystallizable Fragment Glycoengineering for Therapeutic Antibodies Development. Front. Immunol..

[58] Umana P., Jean-Mairet J., Moudry R., Amstutz H., Bailey J.E. (1999). Engineered glycoforms of an antineuroblastoma IgG1 with optimized antibody-dependent cellular cytotoxic activity. Nat. Biotechnol..

[59] Yu X., Marshall M.J.E., Cragg M.S., Crispin M. (2017). Improving Antibody-Based Cancer Therapeutics Through Glycan Engineering. BioDrugs.

[60] Peschke B., Keller C.W., Weber P., Quast I., Lunemann J.D. (2017). Fc-Galactosylation of Human Immunoglobulin Gamma Isotypes Improves C1q Binding and Enhances Complement-Dependent Cytotoxicity. Front. Immunol..

[61] Fang J., Richardson J., Du Z., Zhang Z. (2016). Effect of Fc-Glycan Structure on the Conformational Stability of IgG Revealed by Hydrogen/Deuterium Exchange and Limited Proteolysis. Biochemistry.

[62] Kiyoshi M., Tsumoto K., Ishii-Watabe A., Caaveiro J.M.M. (2017). Glycosylation of IgG-Fc: A molecular perspective. Int. Immunol..

[63] Lee H.S., Im W. (2017). Effects of N-Glycan Composition on Structure and Dynamics of IgG1 Fc and Their Implications for Antibody Engineering. Sci. Rep..

[64] Liu S.D., Chalouni C., Young J.C., Junttila T.T., Sliwkowski M.X., Lowe J.B. (2015). Afucosylated antibodies increase activation of FcgammaRIIIa-dependent signaling components to intensify processes promoting ADCC. Cancer Immunol. Res..

[65] Rogers K.A., Huang Y., Ruppert A.S., Awan F.T., Heerema N.A., Hoffman C., Lozanski G., Maddocks K.J., Moran M.E., Reid M.A. (2018). Phase 1b study of obinutuzumab, ibrutinib, and venetoclax in relapsed and refractory chronic lymphocytic leukemia. Blood.

[66] Alpdogan O., Kartan S., Johnson W., Sokol K., Porcu P. (2019). Systemic therapy of cutaneous T-cell lymphoma (CTCL). Chin. Clin. Oncol..

[67] Strohl W.R. (2009). Optimization of Fc-mediated effector functions of monoclonal antibodies. Curr. Opin. Biotechnol..

[68] Kontermann R.E., Brinkmann U. (2015). Bispecific antibodies. Drug Discov. Today.

[69] Schneider-Merck T., Lammerts van Bueren J.J., Berger S., Rossen K., van Berkel P.H., Derer S., Beyer T., Lohse S., Bleeker W.K., Peipp M. (2010). Human IgG2 antibodies against epidermal growth factor receptor effectively trigger antibody-dependent cellular cytotoxicity but, in contrast to IgG1, only by cells of myeloid lineage. J. Immunol..

[70] Kinder M., Greenplate A.R., Strohl W.R., Jordan R.E., Brezski R.J. (2015). An Fc engineering approach that modulates antibody-dependent cytokine release without altering cell-killing functions. MAbs.

[71] Dillon T.M., Ricci M.S., Vezina C., Flynn G.C., Liu Y.D., Rehder D.S., Plant M., Henkle B., Li Y., Deechongkit S. (2008). Structural and functional characterization of disulfide isoforms of the human IgG2 subclass. J. Biol. Chem..

[72] Liu H., May K. (2012). Disulfide bond structures of IgG molecules: Structural variations, chemical modifications and possible impacts to stability and biological function. MAbs.

[73] White A.L., Chan H.T., French R.R., Willoughby J., Mockridge C.I., Roghanian A., Penfold C.A., Booth S.G., Dodhy A., Polak M.E. (2015). Conformation of the human immunoglobulin G2 hinge imparts superagonistic properties to immunostimulatory anticancer antibodies. Cancer Cell.

[74] Brezski R.J., Oberholtzer A., Strake B., Jordan R.E. (2011). The in vitro resistance of IgG2 to proteolytic attack concurs with a comparative paucity of autoantibodies against peptide analogs of the IgG2 hinge. MAbs.

[75] Brezski R.J., Kinder M., Grugan K.D., Soring K.L., Carton J., Greenplate A.R., Petley T., Capaldi D., Brosnan K., Emmell E. (2014). A monoclonal antibody against hinge-cleaved IgG restores effector function to proteolytically-inactivated IgGs in vitro and in vivo. MAbs.

[76] Aalberse R.C., Schuurman J. (2002). IgG4 breaking the rules. Immunology.

[77] Labrijn A.F., Buijsse A.O., van den Bremer E.T., Verwilligen A.Y., Bleeker W.K., Thorpe S.J., Killestein J., Polman C.H., Aalberse R.C., Schuurman J. (2009). Therapeutic IgG4 antibodies engage in Fab-arm exchange with endogenous human IgG4 in vivo. Nat. Biotechnol..

[78] van der Neut Kolfschoten M., Schuurman J., Losen M., Bleeker W.K., Martinez-Martinez P., Vermeulen E., den Bleker T.H., Wiegman L., Vink T., Aarden L.A. (2007). Anti-inflammatory activity of human IgG4 antibodies by dynamic Fab arm exchange. Science.

[79] Angal S., King D.J., Bodmer M.W., Turner A., Lawson A.D., Roberts G., Pedley B., Adair J.R. (1993). A single amino acid substitution abolishes the heterogeneity of chimeric mouse/human (IgG4) antibody. Mol. Immunol..

[80] Stapleton N.M., Andersen J.T., Stemerding A.M., Bjarnarson S.P., Verheul R.C., Gerritsen J., Zhao Y., Kleijer M., Sandlie I., de Haas M. (2011). Competition for FcRn-mediated transport gives rise to short half-life of human IgG3 and offers therapeutic potential. Nat. Commun..

[81] Hatjiharissi E., Xu L., Santos D.D., Hunter Z.R., Ciccarelli B.T., Verselis S., Modica M., Cao Y., Manning R.J., Leleu X. (2007). Increased natural killer cell expression of CD16, augmented binding and ADCC activity to rituximab among individuals expressing the FcγRIIIa-158 V/V and V/F polymorphism. Blood.

[82] Capuano C., Pighi C., Molfetta R., Paolini R., Battella S., Palmieri G., Giannini G., Belardinilli F., Santoni A., Galandrini R. (2017). Obinutuzumab-mediated high-affinity ligation of FcgammaRIIIA/CD16 primes NK cells for IFNgamma production. Oncoimmunology.

[83] Horikawa M., Minard-Colin V., Matsushita T., Tedder T.F. (2011). Regulatory B cell production of IL-10 inhibits lymphoma depletion during CD20 immunotherapy in mice. J. Clin. Investig..

[84] Oflazoglu E., Stone I.J., Gordon K.A., Grewal I.S., van Rooijen N., Law C.L., Gerber H.P. (2007). Macrophages contribute to the antitumor activity of the anti-CD30 antibody SGN-30. Blood.

[85] Oflazoglu E., Stone I.J., Brown L., Gordon K.A., van Rooijen N., Jonas M., Law C.L., Grewal I.S., Gerber H.P. (2009). Macrophages and Fc-receptor interactions contribute to the antitumour activities of the anti-CD40 antibody SGN-40. Brit. J. Cancer.

[86] Gul N., Babes L., Siegmund K., Korthouwer R., Bogels M., Braster R., Vidarsson G., ten Hagen T.L., Kubes P., van Egmond M. (2014). Macrophages eliminate circulating tumor cells after monoclonal antibody therapy. J. Clin. Investig..

[87] Dahal L.N., Dou L., Hussain K., Liu R., Earley A., Cox K.L., Murinello S., Tracy I., Forconi F., Steele A.J. (2017). STING Activation Reverses Lymphoma-Mediated Resistance to Antibody Immunotherapy. Cancer Res..

[88] Nagelkerke S.Q., Bruggeman C.W., den Haan J.M.M., Mul E.P.J., van den Berg T.K., van Bruggen R., Kuijpers T.W. (2018). Red pulp macrophages in the human spleen are a distinct cell population with a unique expression of Fc-gamma receptors. Blood Adv..

[89] Martinez F.O., Sica A., Mantovani A., Locati M. (2008). Macrophage activation and polarization. Front. Biosci..

[90] Kang T.H., Lee C.H., Delidakis G., Jung J., Richard-Le Goff O., Lee J., Kim J.E., Charab W., Bruhns P., Georgiou G. (2019). An Engineered Human Fc variant With Exquisite Selectivity for FcgammaRIIIaV158 Reveals That Ligation of FcgammaRIIIa Mediates Potent Antibody Dependent Cellular Phagocytosis With GM-CSF-Differentiated Macrophages. Front. Immunol..

[91] Teige I., Martensson L., Frendeus B.L. (2019). Targeting the Antibody Checkpoints to Enhance Cancer Immunotherapy-Focus on FcgammaRIIB. Front. Immunol..

[92] DiLillo D.J., Ravetch J.V. (2015). Differential Fc-Receptor Engagement Drives an Anti-tumor Vaccinal Effect. Cell.

[93] Postow M.A., Chesney J., Pavlick A.C., Robert C., Grossmann K., McDermott D., Linette G.P., Meyer N., Giguere J.K., Agarwala S.S. (2015). Nivolumab and ipilimumab versus ipilimumab in untreated melanoma. N. Engl. J. Med..

[94] Roghanian A., Teige I., Martensson L., Cox K.L., Kovacek M., Ljungars A., Mattson J., Sundberg A., Vaughan A.T., Shah V. (2015). Antagonistic human FcgammaRIIB (CD32B) antibodies have anti-tumor activity and overcome resistance to antibody therapy in vivo. Cancer Cell.

[95] Beers S.A., French R.R., Chan H.T., Lim S.H., Jarrett T.C., Vidal R.M., Wijayaweera S.S., Dixon S.V., Kim H., Cox K.L. (2010). Antigenic modulation limits the efficacy of anti-CD20 antibodies: Implications for antibody selection. Blood.

[96] Lim S.H., Vaughan A.T., Ashton-Key M., Williams E.L., Dixon S.V., Chan H.T., Beers S.A., French R.R., Cox K.L., Davies A.J. (2011). Fc gamma receptor IIb on target B cells promotes rituximab internalization and reduces clinical efficacy. Blood.

[97] Camilleri-Broet S., Cassard L., Broet P., Delmer A., Le Touneau A., Diebold J., Fridman W.H., Molina T.J., Sautes-Fridman C. (2004). FcgammaRIIB is differentially expressed during B cell maturation and in B-cell lymphomas. Br. J. Haematol..

[98] Johnson L.S., Huang L., Gerena R. (2019). FcgammaRIIB-Specific Antibodies and Methods of Use Thereof. U.S. Patent.

[99] Arce Vargas F., Furness A.J.S., Litchfield K., Joshi K., Rosenthal R., Ghorani E., Solomon I., Lesko M.H., Ruef N., Roddie C. (2018). Fc Effector Function Contributes to the Activity of Human Anti-CTLA-4 Antibodies. Cancer Cell.

[100] Arlauckas S.P., Garris C.S., Kohler R.H., Kitaoka M., Cuccarese M.F., Yang K.S., Miller M.A., Carlson J.C., Freeman G.J., Anthony R.M. (2017). In vivo imaging reveals a tumor-associated macrophage-mediated resistance pathway in anti-PD-1 therapy. Sci. Transl. Med..

[101] Dahan R., Sega E., Engelhardt J., Selby M., Korman A.J., Ravetch J.V. (2015). FcgammaRs Modulate the Anti-tumor Activity of Antibodies Targeting the PD-1/PD-L1 Axis. Cancer Cell.

[102] Hogarth P.M., Pietersz G.A. (2012). Fc receptor-targeted therapies for the treatment of inflammation, cancer and beyond. Nat. Rev. Drug Discov..

[103] Engblom C., Pfirschke C., Pittet M.J. (2016). The role of myeloid cells in cancer therapies. Nat. Rev. Cancer.

[104] Guilliams M., Bruhns P., Saeys Y., Hammad H., Lambrecht B.N. (2014). The function of Fcgamma receptors in dendritic cells and macrophages. Nat. Rev. Immunol..

[105] Rankin C.T., Veri M.C., Gorlatov S., Tuaillon N., Burke S., Huang L., Inzunza H.D., Li H., Thomas S., Johnson S. (2006). CD32B, the human inhibitory Fc-gamma receptor IIB, as a target for monoclonal antibody therapy of B-cell lymphoma. Blood.

[106] Jung S.T., Reddy S.T., Kang T.H., Borrok M.J., Sandlie I., Tucker P.W., Georgiou G. (2010). Aglycosylated IgG variants expressed in bacteria that selectively bind FcgammaRI potentiate tumor cell killing by monocyte-dendritic cells. Proc. Natl. Acad. Sci. USA.

[107] White A.L., Chan H.T., Roghanian A., French R.R., Mockridge C.I., Tutt A.L., Dixon S.V., Ajona D., Verbeek J.S., Al-Shamkhani A. (2011). Interaction with FcgammaRIIB is critical for the agonistic activity of anti-CD40 monoclonal antibody. J. Immunol..

[108] Li F., Ravetch J.V. (2011). Inhibitory Fcgamma receptor engagement drives adjuvant and anti-tumor activities of agonistic CD40 antibodies. Science.

[109] White A.L., Chan H.T., French R.R., Beers S.A., Cragg M.S., Johnson P.W., Glennie M.J. (2013). FcgammaRIotaIotaB controls the potency of agonistic anti-TNFR mAbs. Cancer Immunol. Immunother..

[110] Beutier H., Hechler B., Godon O., Wang Y., Gillis C.M., de Chaisemartin L., Gouel-Cheron A., Magnenat S., Macdonald L.E., Murphy A.J. (2018). Platelets expressing IgG receptor FcgammaRIIA/CD32A determine the severity of experimental anaphylaxis. Sci. Immunol..

[111] Smith P., DiLillo D.J., Bournazos S., Li F., Ravetch J.V. (2012). Mouse model recapitulating human Fcgamma receptor structural and functional diversity. Proc. Natl. Acad.Sci. USA.

[112] Lee C.H., Kang T.H., Godon O., Watanabe M., Delidakis G., Gillis C.M., Sterlin D., Hardy D., Cogne M., Macdonald L.E. (2019). An engineered human Fc domain that behaves like a pH-toggle switch for ultra-long circulation persistence. Nat. Commun..

